# Supplementary material for: Adenosine Derivates as Antioxidant Agents: Synthesis, Characterization, in Vitro Activity, and Theoretical Insights
Source: Antioxidants (Basel). 2019 Oct 9;8(10):468. doi: 10.3390/antiox8100468 (PMC6826950; doi:10.3390/antiox8100468)

Adenosine derivates as antioxidant agents: synthesis, characterization, *in vitro* activity and theoretical insights.

Francisco Valdes[2], Nelson Brown[3], Alejandro Morales-Bayuelo[4], Luis Prent-Peñaloza[1], Margarita Gutierrez[2]

[1] Organic Synthesis Laboratory and Biological Activity (LSO-Act-Bio), PhD Applied Sciences, faculty of Engineering and Institute of Chemistry of Natural Resources, Universidad de Talca, Casilla 747, Talca 3460000, Chile. luisprent@gmail.com

[2] Organic Synthesis Laboratory and Biological Activity (LSO-Act-Bio), PhD sciences mention investigation and development of bioactive products, Institute of Chemistry of Natural Resources, Universidad de Talca, Casilla 747, Talca 3460000, Chile. franciscovaldesz@hotmail.es

[3] Center for Medical Research, University of Talca School of Medicine, 3460000 Talca, Chile; Programa de Investigation Asociativa en Cancer Gastrico (PIA-CG), Universidad de Talca. nbrown@utalca.cl

[4] Centro de Investigación de Procesos del Tecnológico Comfenalco (CIPTEC), programa de Ingeniería Industrial, Fundación Universitaria Tecnológico Comfenalco – Cartagena, Cr 44 D N 30A, 91, Cartagena-Bolívar, 130001Colombia. alejandromoraba@hotmail.com

SUPPLEMENTARY MATERIAL

CONTENTS

Figure S1. IR, ^{1}H and ^{13}C nuclear magnetic resonance (NMR) spectra of compound **1a**…….**3**

Figure S2. IR, ^{1}H and ^{13}C nuclear magnetic resonance (NMR) spectra of compound **1b**…….**4**

Figure S3. IR, ^{1}H and ^{13}C nuclear magnetic resonance (NMR) spectra of compound **1c**……..**5**

Figure S4. IR, ^{1}H and ^{13}C nuclear magnetic resonance (NMR) spectra of compound **2**…........**6**

Figure S5. IR, ^{1}H and ^{13}C nuclear magnetic resonance (NMR) spectra of compound **3**……....**7**

Figure S6. IR, ^{1}H and ^{13}C nuclear magnetic resonance (NMR) spectra of compound **4**……....**8**

Figure S7. IR, ^{1}H and ^{13}C nuclear magnetic resonance (NMR) spectra of compound **5**……....**9**

Figure S1. Compound 1a: *2-(6-butylamino-purin-9-yl]-5-hydroxymethyl-tetrahydro-furan-3,4-diol*

IR (KBr):

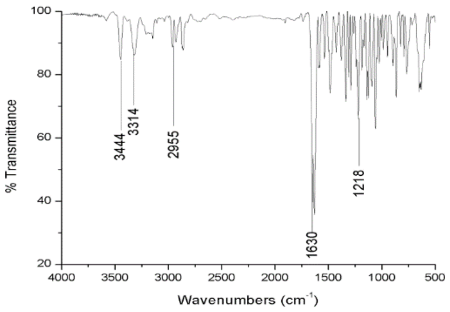

^{1}H-RMN (DMSO-d_6, 400MHz)

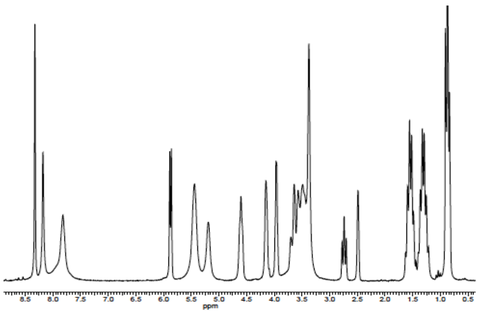

^{13}C-RMN (DMSO-d_6, 100 MHz)

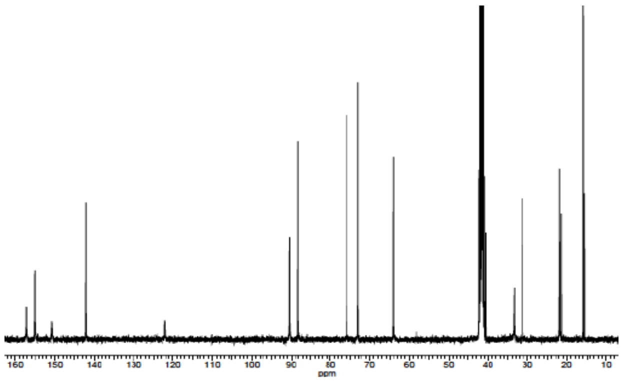

Figure S2. Compound 1b: *2-[6-(5-amino-2-methyl-pentylamino)-purin-9-yl]-5-hydroxymethyl-tetrahydro-furan-3,4-diol*

IR (KBr):

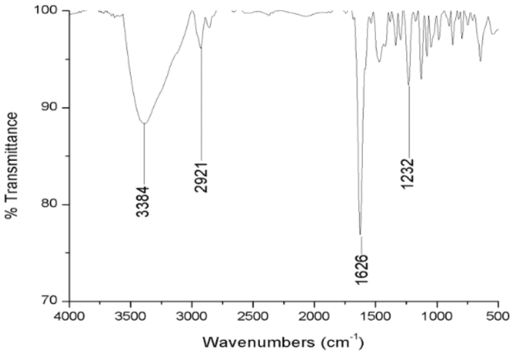

^{1}H-RMN (DMSO-d_6, 400 MHz)

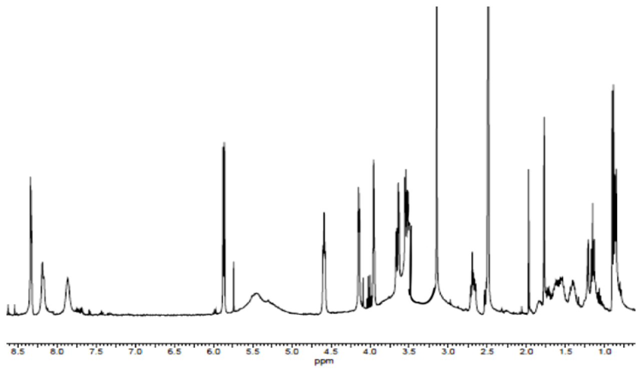

^{13}C-RMN (DMSO-d_6, 100 MHz)

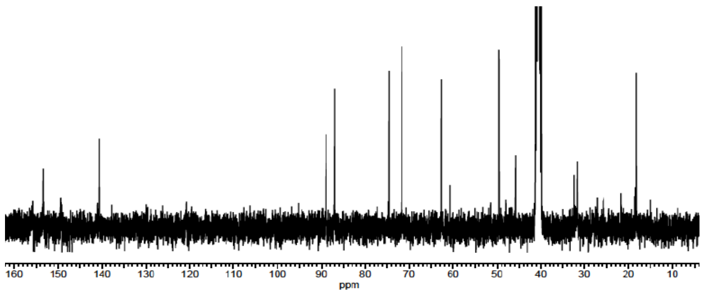

Figure S3. Compound 1c: *2-[6-(3-propylamino)-purin-9-yl]-5-hydroxymethyl-tetrahydro-furan-3,4-diol*

IR (KBr):

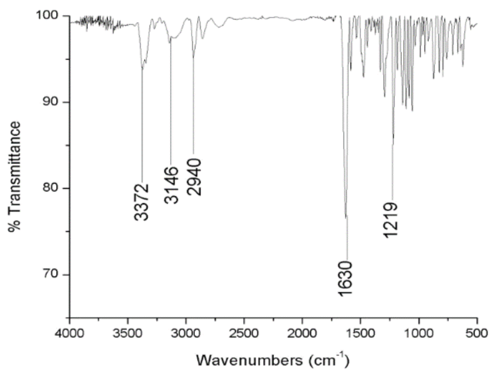

^{1}H-RMN (DMSO-d_6, 400 MHz)

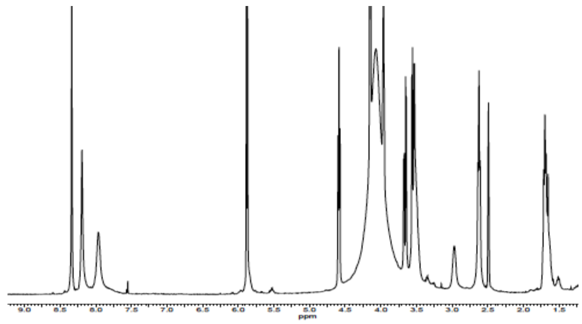

^{13}C-RMN (DMSO-d_6, 100 MHz)

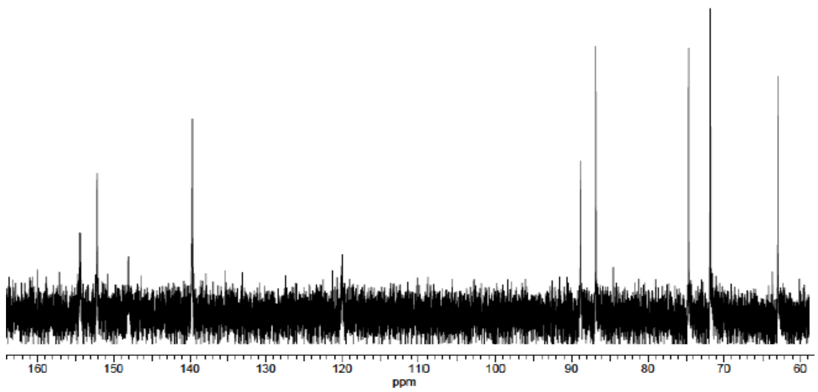

Figure S4. Compound 2: *[6-(6-Chloro-purin-9-yl)-2,2-dimethyl-tetrahydro-furo[3,4-d][1,3]dioxol-4-yl]-methanol*

IR (KBr):

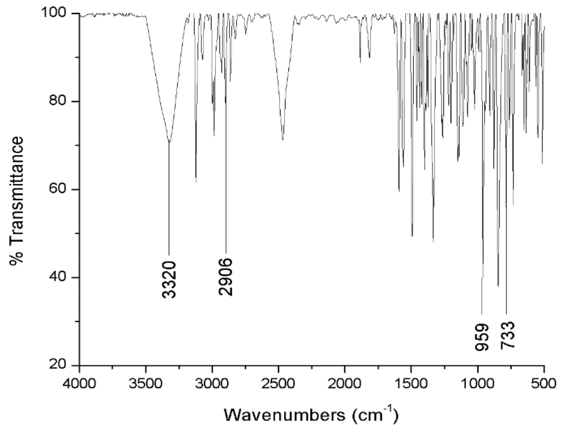

^{1}H-RMN (DMSO-d6, 400 MHz)

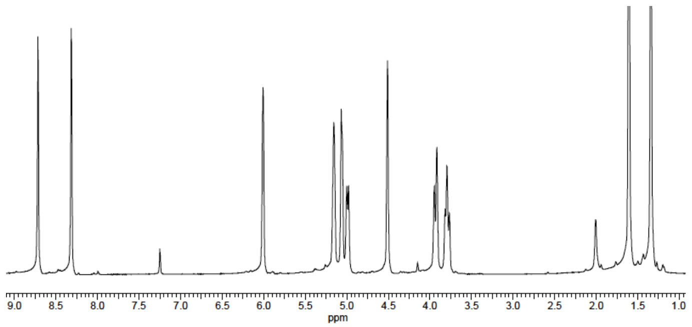

^{13}C-RMN (DMSO-d6, 100 MHz)

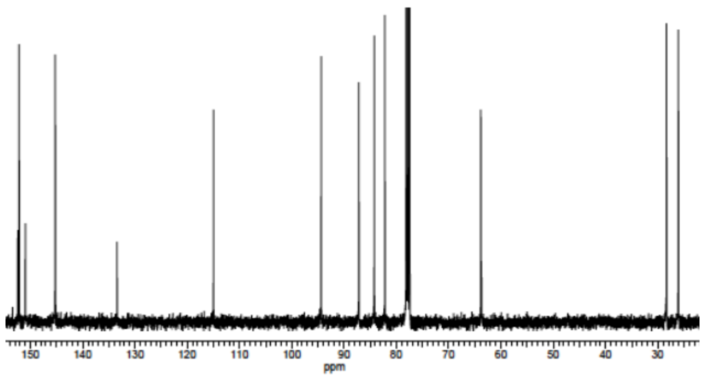

Figure S5. Compound 3: *6-(6-Chloro-purin-9-yl)-2,2-dimethyl-tetrahydro-furo[3,4-d][1,3]dioxole-4-carboxylic acid*

IR (KBr):

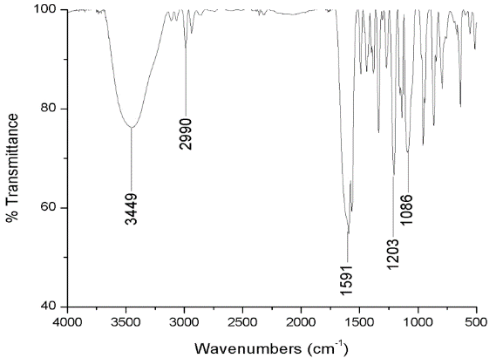

^{1}H-RMN (DMSO-d$_6$, 400 MHz)

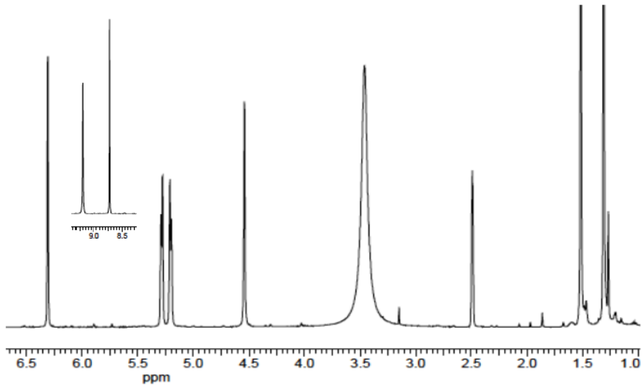

^{13}C-RMN (DMSO-d$_6$, 100 MHz)

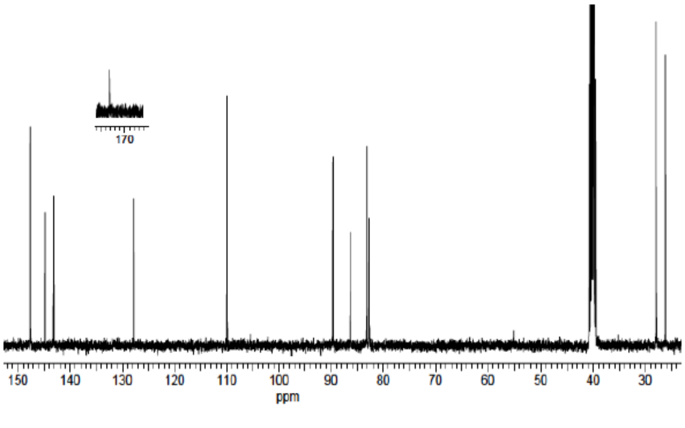

Figure S6. Compound 4: *1′-deoxy-1′-(6-chloro-9H-purin-9-yl) -β-D-ribofuranuronic acid*

IR (KBr):

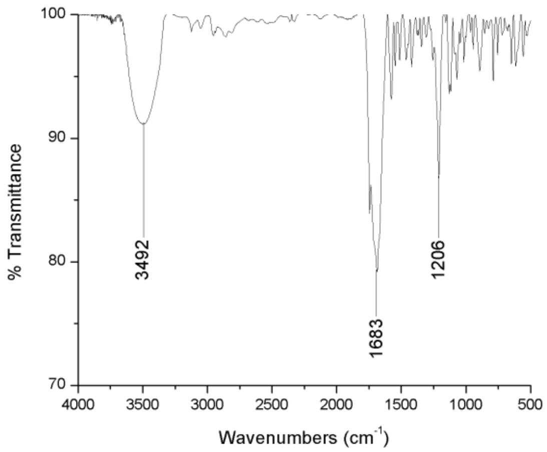

^{1}H-RMN (DMSO-d6, 400 MHz)

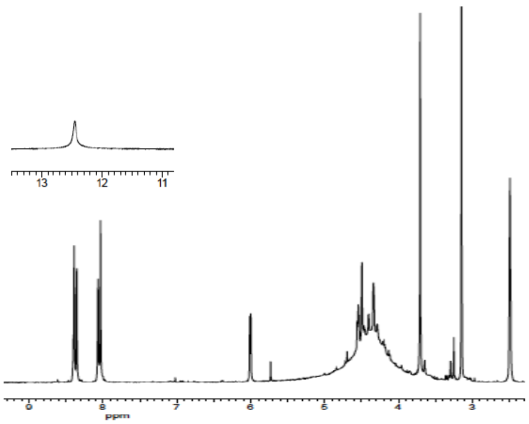

^{13}C-RMN (DMSO-d6, 100 MHz)

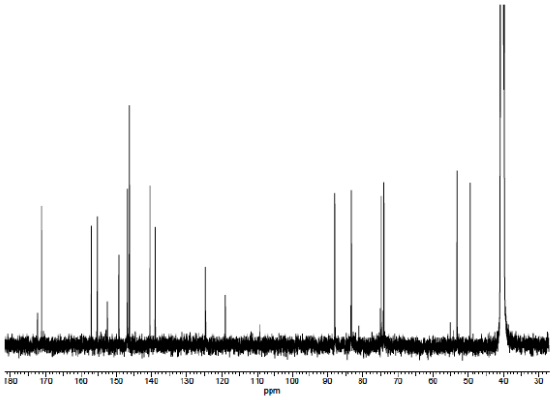

Figure S7. Compound 5: *6 - [(4-amino-2,3,5-triazole) thio] -β-D-ribofuranosyl-9H-purine-5′-N-ethyluronamide*

IR (KBr):

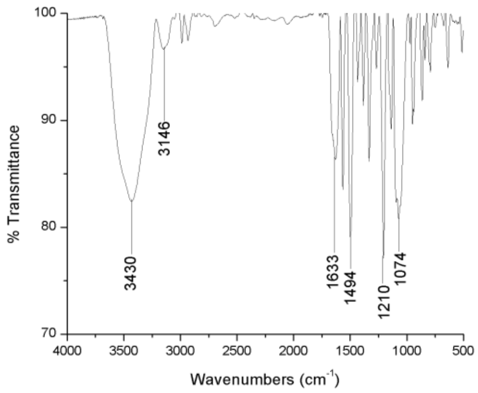

^{1}H-RMN (DMSO-d$_6$, 400 MHz)

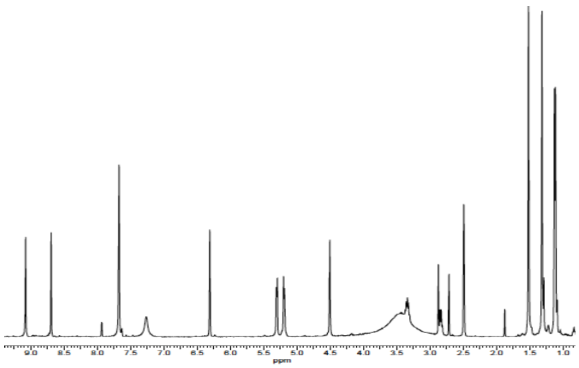

^{13}C-RMN (DMSO-d$_6$, 100 MHz)

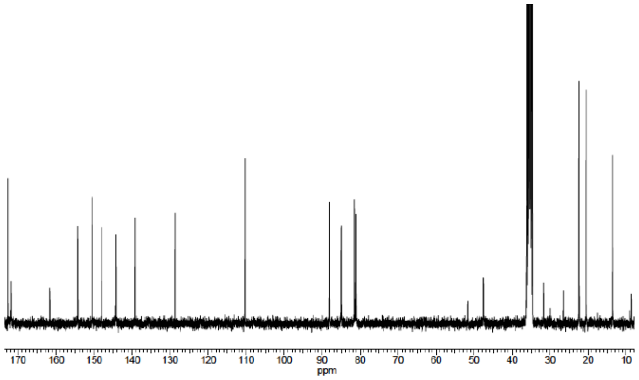

Supplement: Supplementary file 1 [file antioxidants-08-00468-s001.pdf]
